# TET1 promotes fatty acid oxidation and inhibits NAFLD progression by hydroxymethylation of PPARα promoter

**DOI:** 10.1186/s12986-020-00466-8

**Published:** 2020-06-19

**Authors:** Jingjie Wang, Yitong Zhang, Qin Zhuo, Yujen Tseng, Jiucun Wang, Yanyun Ma, Jun Zhang, Jie Liu

**Affiliations:** 1 Department of Digestive Diseases of Huashan Hospital and Institutes of Biomedical Sciences, Fudan University, Shanghai, China; 2 State Key Laboratory of Genetic Engineering, Human Phenome Institute, Fudan University, Shanghai, China; 3 Six-sector Industrial Research Institute, Fudan University, Shanghai, 200433 China

**Keywords:** TET1, NAFLD, PPARα, Fatty acid oxidation, Hydroxymethylation

## Abstract

**Background:**

As a lipid metabolic disorder, non-alcoholic fatty liver disease (NAFLD) is an important cause of cirrhosis and hepatocellular carcinoma, with no effective drug up to date. Previous studies have demonstrated increased methylation levels of key genes in NAFLD, suggesting that hydroxymethylation, a key step in demethylation, may be a possible strategy to reverse NAFLD. TET1 is well known as a key hydroxymethylase, however, its role and mechanism in NAFLD remains unclear.

**Methods:**

In this study, we utilized TET1 knockout mice, fed with high-fat diet. Furthermore, by ChIP and hMeDIP. TET1 knockdown L02 and HepG2 cell lines.

**Results:**

Their degree of liver steatosis was more severe than that of wild-type mice, suggesting that TET1 had a significant protective effect against NAFLD. We further found that PPARα, a key regulator of fatty acid oxidation, and its downstream key enzymes ACOX1 and CPT1A, as well as the fatty acid oxidation product β-HB were significantly decreased in TET1 knockout mice. While the key genes for fatty acid synthesis and uptake were not significantly changed, suggesting that TET1 inhibits NAFLD by promoting fatty acid oxidation via PPARα pathway. TET1 was confirmed to directly bind to the promoter of PPARα and elevate its hydroxymethylation level.

**Conclusions:**

This study is the first to show that TET1 can activate PPARα, promote fatty acid oxidation and inhibit NAFLD progression by hydroxymethylation of PPARα promoter, which may be a new strategy to reverse NAFLD.

## Background

As a metabolic disease derived from lipid disorder, NAFLD is the most common chronic liver disease, which is an important and common cause of liver fibrosis and hepatocellular carcinoma (HCC) [1, 2]. Compared to other etiologies, such as HBV, HCV, and alcohol, NAFLD has become the most stable increasing cause of HCC [3]. Currently, NAFLD and non-alcoholic steatohepatitis (NASH) are considered to be modified by a variety of environmental elements which act on susceptible genes and epigenetic backgrounds, but the specific mechanism is still unclear [4]. At present, there is no government-approved drug for the treatment of NAFLD [5], therefore it is necessary to study the pathogenesis of NAFLD, in pursuit of new therapeutic targets.

The TET protein is a DNA cytosine oxygenase that catalyzes 5-methyl cytosine (5mC) to produce 5-hydroxymethylcytosine (5-hmC) in a manner dependent on α-ketoglutarate (α-KG) and Fe2+ [6]. The mammalian TET protein has three family members, TET1, TET2 and TET3. TET maintains the unmethylated status of genes by the above-described “active” demethylation and the so called “passive” demethylation which is competitive with DNA methyltransferases [6]. Studies have shown that TET and oxidized 5-mC derivatives (such as 5-hmC) play important roles in various biological and pathological processes including gene transcription, embryonic development and tumorigenesis [7, 8]. However, there is a lack of relevant research on the role of TET in metabolic diseases such as NAFLD. Previous studies have revealed that some DNA and mitochondrial DNA are methylated during the pathogenesis of NAFLD [9–11], suggesting that demethylation process may be involved in NAFLD. It has also been shown that missense mutations in the TET1 and TET2 loci are associated with NAFLD and type 2 diabetes [12]. Therefore, it is speculated that TET may play an important role in NAFLD. The present study intends to study the role of TET genes in lipid metabolism and pathogenesis of NAFLD, explore the target and specific mechanism, and provide new ideas for the diagnosis and treatment of NAFLD.

## Methods

### Animals

The animal protocol was approved by the institutional Animal Care and Use Committee at the Fudan University. The C57BL/6 background TET1 knockout heterozygote (HE) mouse was purchased from the Jackson Laboratory, and the WT and TET1 −/− mice genotypes were determined by PCR using following primers: wild type Forward: TCAGGGAGCTCATGGAGACTA; Common: TTAAAGCATGGGTGGGAGTC; Mutant Forward: AACTGATTCCCTTCGTGCAG. Homozygous (HO) has a band at 650 bp, and HE has a band at both 650 bp and 300 bp, WT has a band at 300 bp. Only male mice were used in these studies. Male mice of 8–10 weeks old (about 25 g) were used. The mice in the NAFLD group were fed a high-fat diet (HFD; carbohydrates, 20.3%; protein, 18.1%; fat, 61.6%; D12492, Research Diets, New Brunswick, NJ, USA) for 12 weeks. The mice in the control group were fed normal chow (NC; carbohydrates, 71.5%; protein, 18.3%; fat, 10.2%; D12450B, Research Diets) for 12 weeks. There are 6 mice in each group.

### Glucose tolerance test (GTT) and insulin tolerance test (ITT)

GTT and ITT experiments were performed at 10 and 11 weeks after feeding HFD or normal food. Mice were fasted overnight, and the next morning, 1 g/kg of glucose or 0.75 U/kg of insulin was administered intraperitoneally. Blood glucose was measured at 0, 15, 30, 60, and 120 min after the injection.

### Detection of biochemical indicators in plasma

Plasma triglyceride (TG), total cholesterol (TC), lipoprotein, AST and ALT were detected by automated biochemical analyzer. Plasma insulin detection with Mouse Insulin ELISA Kit (Crystal Chem, IL, USA) was used according to instructions. The end point calorimetric assays were performed using a BioTek PowerWave XS Microplate spectrophotometer.

### Detection of liver tissue and intracellular triglyceride

Intrahepatic and intracellular triglyceride (TG) contents were assayed using kits purchased from Applygen Technologies Inc. (Beijing, China) in accordance with the vendor’s recommended protocols. Briefly, the lysis of cells and tissues with lysate were assayed for total protein concentration using a BCA Protein Assay Kit (Thermo Fisher Scientific, MA, USA), and the TG concentration was measured using the reagents in the kit. Finally, the TG content was calculated from the TG concentration to total protein concentration.

### Cell culture and treatment

HpG2 and L02 cells were incubated with DMEM medium (Gibco, Thermo Fisher Scientific, MA, USA) containing 10% fetal bovine serum in a 37 °C incubator containing 5% CO2. The in vitro model of NAFLD was cultured in a medium containing 1 mM oleic acid and palmitic acid (2,1) (Sigma-Aldrich, Munich, Germany) for 24 h. The cells were treated with TET1 plasmid or siTET1 for 24 h and then cultured with FFA-containing medium.

### Tissue and immunofluorescence analysis

H&E and oil red O staining were performed in liver tissue. Liver fat accumulation was observed under a microscope (Olympus, Tokyo, Japan). The cells were incubated with TET1 antibody (11000) (GeneTex, CA, USA) for 2 h at room temperature and then incubated with secondary antibody and fat fluorescent dye BODIPY493/503 (Thermo Fisher Scientific, MA, USA) for 1 h, and the nuclei were stained with DAPI (Beyotime shanghai, China) for 1 min. Results were observed under a Nikon inverted fluorescence microscope (Nikon, Tokyo, Japan).

### Quantitative RT-PCR assay

Total RNA was extracted with TRIzol reagent (Invitrogen, Thermo Fisher Scientific, MA, USA), then reverse transcribed into cDNA using a reverse transcription kit (life technologies- Thermo Fisher Scientific, MA, USA). PCR was amplified with SYBR Green PCR Master Mix (QIAGEN, NRW, Germany). The mRNA expression of the target gene is normalized to β-actin.

*Tet1*(M) -F: ACACAGTGGTGCTAATGCAG.

*Tet1*(M) -R: AGCATGAACGGGAGAATCGG.

*Tet2*(M) -F: AGAGAAGACAATCGAGAAGTCGG.

*Tet2*(M) -R: CCTTCCGTACTCCCAAACTCAT.

*Tet3*(M) -F: TGCGATTGTGTCGAACAAATAGT.

*Tet3*(M) -R: TCCATACCGATCCTCCATGAG.

*Srebp1*(M) -F: CAAGGCCATCGACTACATCCG.

*Srebp1*(M) -R: CACCACTTCGGGTTTCATGC.

*Pparγ*(M) -F: GGAAGACCACTCGCATTCCTT.

*Pparγ*(M) -R: GTAATCAGCAACCATTGGGTCA.

*Fas*(M) -F: GGAGGTGGTGATAGCCGGTAT.

*Fas*(M) -R: TGGGTAATCCATAGAGCCCAG.

*Acc*(M) -F: CTCCCGATTCATAATTGGGTCTG.

*Acc*(M) -R: TCGACCTTGTTTTACTAGGTGC.

*Cd36*(M) -F: ATGGGCTGTGATCGGAACTG.

*Cd36*(M) -R: TTTGCCACGTCATCTGGGTTT.

*Fatp1*(M) -F: TCTGTTCTGATTCGTGTTCGG.

*Fatp1*(M) -R: CAGCATATACCACTACTGGCG.

*Fabp1*(M) -F: ATGAACTTCTCCGGCAAGTACC.

*Fabp1*(M) -R: CTGACACCCCCTTGATGTCC.

*Ppar α*(M) -F: AACATCGAGTGTCGAATATGTGG.

*Ppar α*(M) -R: CCGAATAGTTCGCCGAAAGAA.

*Cpt1a*(M) -F: TGGCATCATCACTGGTGTGTT.

*Cpt1a*(M) -R: GTCTAGGGTCCGATTGATCTTTG.

*Acox1*(M) -F: TAACTTCCTCACTCGAAGCCA.

*Acox1*(M) -R: AGTTCCATGACCCATCTCTGTC.

*β-actin*(M) -F: GGCTGTATTCCCCTCCATCG.

*β-actin*(M) -R: CCAGTTGGTAACAATGCCATGT.

TET1(H) -F: TAATGGAAGCACTGTGGTTTGT.

TET1(H) -R: GCCCCAGATTTGATCTTGGC.

TET2(H) -F: ATACCCTGTATGAAGGGAAGCC.

TET2(H) -R: CTTACCCCGAAGTTACGTCTTTC.

TET3(H) -F: TCCAGCAACTCCTAGAACTGAG.

TET3(H) -R: AGGCCGCTTGAATACTGACTG.

SREBF1(H) -F: ACGGCAGCCCCTGTAACGACCACTGTGA.

SREBF1(H) -R: TGCCAAGATGGTTCCGCCACTCACCAGG.

PPARγ(H) -F: GGGATCAGCTCCGTGGATCT.

PPARγ(H) -R: TGCACTTTGGTACTCTTGAAGTT.

FAS(H) -F: AAGGACCTGTCTAGGTTTGATGC.

FAS(H) -R: TGGCTTCATAGGTGACTTCCA.

ACC(H) -F: ATGTCTGGCTTGCACCTAGTA.

ACC(H) -R: CCCCAAAGCGAGTAACAAATTCT.

PPARα(H) -F: ATGGTGGACACGGAAAGCC.

PPARα(H) -R: CGATGGATTGCGAAATCTCTTGG.

CPT1A(H) -F: TCCAGTTGGCTTATCGTGGTG.

CPT1A(H) -R: TCCAGAGTCCGATTGATTTTTGC.

ACOX1(H) -F: GGCGCATACATGAAGGAGACCT.

ACOX1(H)-R:AGGTGAAAGCCTTCAGTCCAGC.

CD36(H)-F:CAGGTCAACCTATTGGTCAAGCC.

CD36(H)-R:GCCTTCTCATCACCAATGGTCC.

FATP1(H)-F:TGACAGTCGTCCTCCGCAAGAA.

FATP1(H)-R:CTTCAGCAGGTAGCGGCAGATC.

FABP1(H)-F:GTGTCGGAAATCGTGCAGAAT.

FABP1(H)-R:GACTTTCTCCCCTGTCATTGTC.

β-Actin(H) -F: CATGTACGTTGCTATCCAGGC.

β-Actin(H) -R: CTCCTTAATGTCACGCACGAT.

### Western blotting

Cells and tissues were lysed using a mixture containing RIPA Lysis Buffer (Beyotime shanghai, China) and Protease inhibitor cocktail (Beyotime shanghai, China), and then shaken for uniform mixture. The protein-containing supernatant was centrifuged, then separated by SDS-PAGE and transferred to the PVDF membrane (Millipore) by electrophoretic transfer. After blocking with 5% bovine serum albumin, the membrane was incubated overnight with a diluted primary antibody at 4 °C. After repeated rinsing, the membrane was incubated with the corresponding secondary antibody at 37 °C at a 1:5000 dilution for 1 h.

#### Primary antibody

TET1(GeneTex, CA, USA), PPARα (Abcam, Camb,UK), ACOX1(Proteintech, IL, USA), CPT1A(Proteintech, IL, USA), CD36(Abcam, Camb,UK). β-actin((Proteintech, IL, USA)).

#### Secondary antibody

Horseradish peroxidase (HRP)-conjugated anti-rabbit and anti- mouse (Beyotime shanghai, China).

Proteins were extracted from cells using RIPA Lysis Buffer (Beyotime) with Protease inhibitor cocktail for general use (Beyotime), separated by SDS-PAGE, and electrophoretically transferred to PVDF membranes (Millipore, MA, USA).

### ChIP assay

ChIP assay was carried out using the ChIP assay kit (Chromatin Immunoprecipitation Assay kit, Millipore, MA, USA) according to protocol. Briefly, 1 × 10^7^ cells were harvested and treated with 1% formaldehyde for 10 min at 37 °C to cross-link. Glycine was added to the cell suspension such that its final concentration was 0.125 M to terminate the reaction. The chromatin was sheared on ice by sonication to produce DNA fragments of 200 to 1000 bp. After centrifugation, the cell lysates were incubated with indicated antibody overnight and subsequently with protein G-agarose beads for 2 ~ 4 h at 4 °C with agitation. Beads were washed and eluted, and the cross links were reversed by incubation at 65 °C for 4 h. Finally, the beads were washed and eluted, and the cross-linking was reversed by incubation at 65 °C for 4 h. The purified DNA was used to analyze the binding of TET1 to the PPARα promoter locus by Q-ChIP reaction. The sequences of oligonucleotides were used as Q-ChIP primers:

− 1017——-717 F: CGCTCCGGCAGCTCGAGCGTCACGG.

− 1017——-717 R: TGAGGGGCTGACTTTGTGCCTACGC.

− 716——-416 F: GCCACCTGTTTCCTTGTCCTCCCAG.

− 716——-416 R: GAGGCTCAGAAGTGCGTAGGGTGGG.

− 415——-115 F: GAGGGGCGCTGACGCTCAGCGGTGT.

− 415——-115 R: TCAGCGGCTCCCACCTAGCG.

### hMeDIP-Q-PCR

The 5hmC status of DNA of normal and siTET1 cell samples was determined by hMeDIP. In summary, genomic DNA was extracted and sheared by sonication into 200–1000 bp of fragments, and the DNA solution containing 5hmC was extracted according to the hMeDIP kit (EpiQuik Hydroxymethylated DNA Immunoprecipitation Kit, Epigentek, NY, USA) protocol. After the sample was placed in the sample plate, Non-immune IgG was added to the negative control group, while 5-hmC Antibody was added to the sample well and the positive control group. After incubation for 60 min at room temperature, each well was washed sequentially with different solutions. Finally, the prepared DRB-PK reagent was added to the well and placed in a 65 °C environment for 20 min, and the DNA-containing solution was taken up for subsequent Q-PCR. The precipitated DNA was used for Q-PCR by using the following primers:

− 415——-115 F: GAGGGGCGCTGACGCTCAGCGGTGT.

− 415——-115 R: TCAGCGGCTCCCACCTAGCG.

## Results

### Decreased expression of TET1 in NAFLD models in vitro and in vivo

**Fig. 1:**
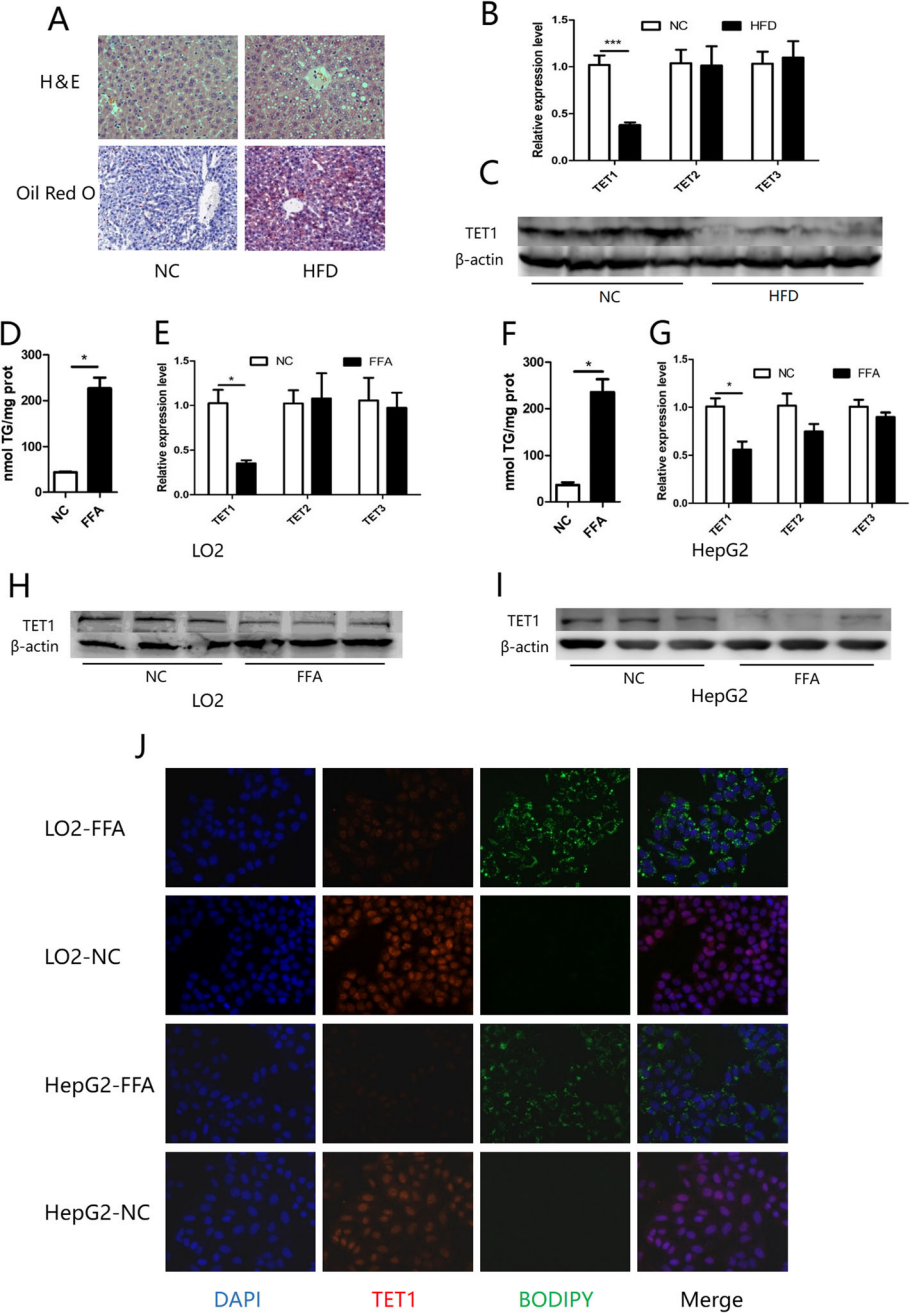
Wild-type mice were fed with HFD or normal diet for 12 weeks, and liver change (**a**) was observed by H&E and oil red O staining. The TET family was detected by Q-PCR after total mRNA was extracted from the liver (**b**). After stimulating the cell steatosis with FFA-containing medium, the intracellular TG content was measured (**d,f**). The mRNA of the TET family in the cells was detected by a Q-PCR method, and normalized to β-actin (**e,g**). The content of TET1 in mouse liver and cells was detected by WB method (**c,h,i**). (* *p* < 0.05, *** *p* < 0.001). Intracellular lipid droplets BODIPY 493/503 (green) DNA stained with DAPI (blue), TET1 (red) (**j**)

In order to explore the role of the TET family in lipid metabolism and fatty liver, wild type (WT) mice were fed with a high-fat diet (HFD) for 12 weeks in order to establish a mouse NAFLD model (Fig. 1 a). Compared with normal liver, the mRNA level of TET1 in fatty liver was significantly reduced, while changes in TET2 and TET3 were not significant (Fig. 1 b). In addition, TET1 also showed a significant decrease in protein level in fatty liver (Fig. 1 c). To further confirm our findings, we explored the in vitro model of hepatic steatosis, which stimulates L02 and HepG2 cell steatosis with oleic acid and palmitic acid (Fig. 1 d, f, j). Compared with the control group, the mRNA and protein levels of TET1 in the two steatosis cell lines were significantly decreased, while the mRNAs of TET2 and TET3 were not significantly changed (Fig. 1 e, g, h, i, j).

### The knockout of TET1 can exacerbate HFD-induced fatty liver

**Fig. 2:**
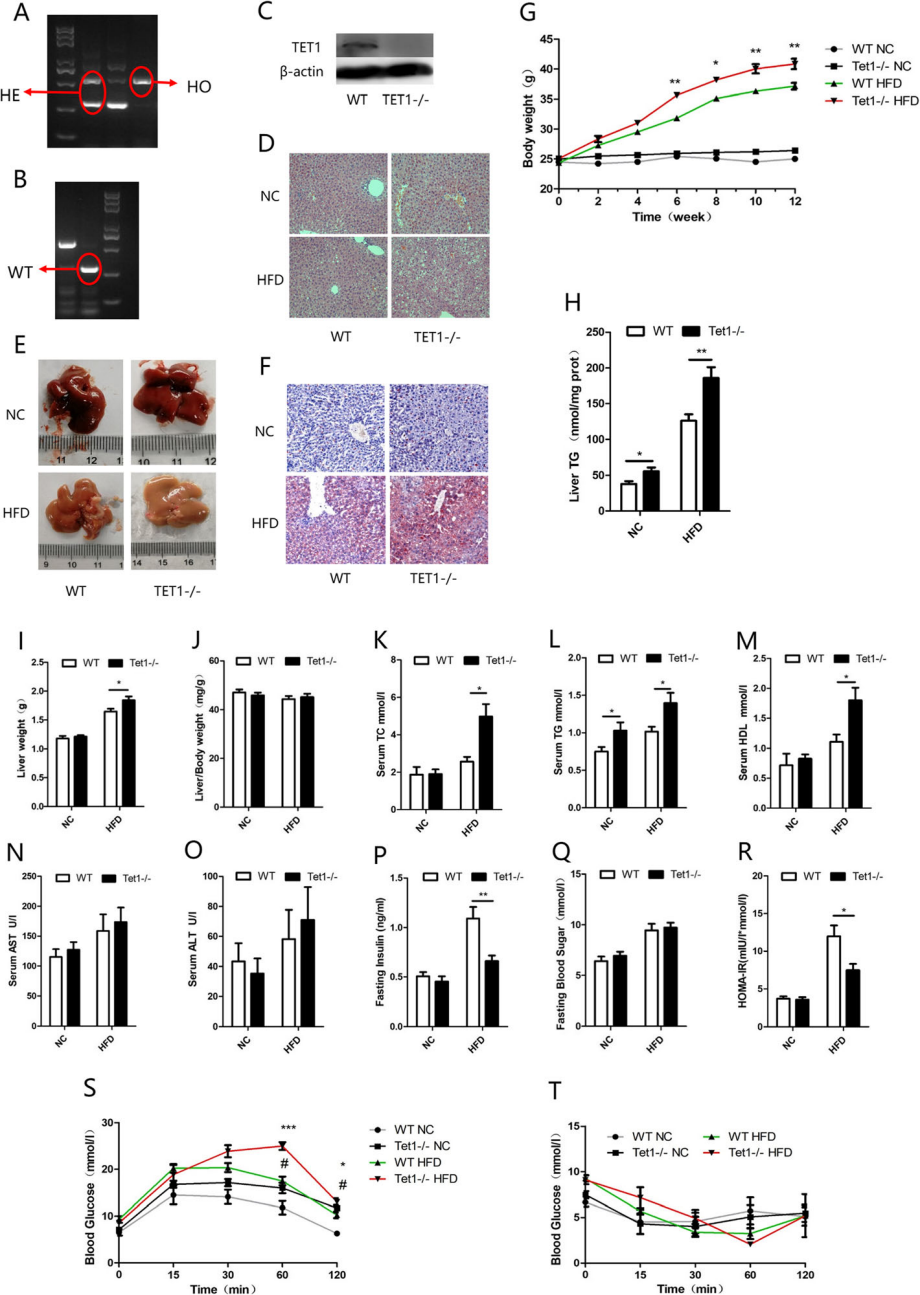
After DNA extraction, mice were subjected to PCR and gel electrophoresis (**a**). Compared with WT mice, lipid accumulation in the liver of TET1 knockout mouse was compared in gross view of the liver (**e**), liver weight (**i**), body weight (**g**), liver index (**j**), H&E (**d**), Oil red O staining (**f**), Comparison of TG content in the liver (**h**). The levels of ALT (**o**), and AST (**n**) in the serum, and fasting blood sugar (**q**), fasting insulin (**p**), and HOMA-IR (**r**) values of TET1-KO mice and their littermate controls at 0–12 weeks after NC or HFD feeding. The blood glucose levels of WT and TET1-KO mice according to glucose tolerance test (GTT) (**s**) and insulin tolerance test (ITT) (**t**) at 11 weeks after HFD treatment (*n* = 6 mice in each group for each test)

**Fig. 3:**
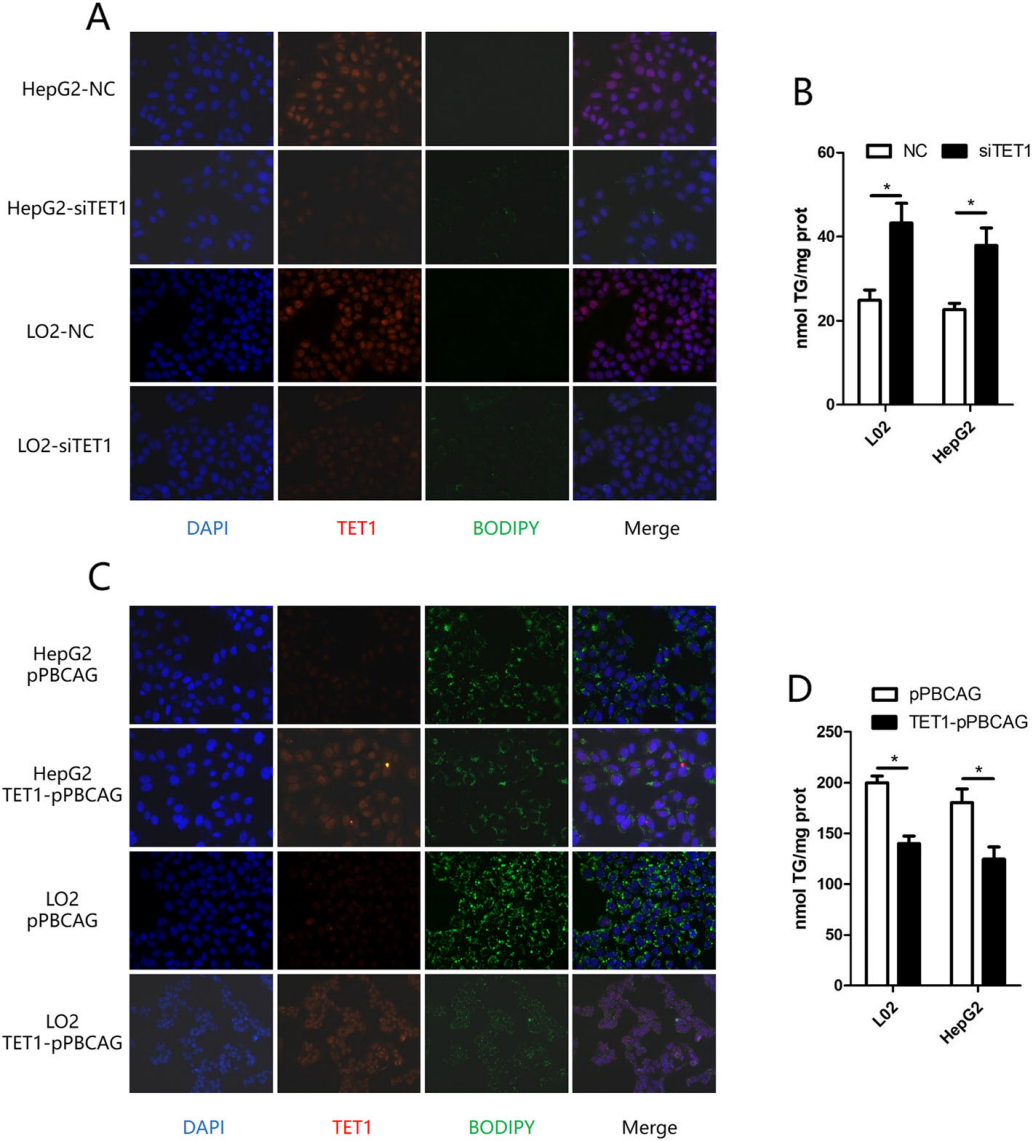
After transfection of siTET1 and negative control, immunofluorescence of TG and TET1 in hepatocytes, lipid drop BODIPY 493/503 (green) DNA stained with DAPI (blue), TET1 (red) (**a**), and intracellular TG content were compared (**b**). Transfection of TET1 expression plasmid (TET1-pPBCAG) and control plasmid (pPBCAG), immunofluorescence of TG and TET1 in hepatocytes, lipid drop BODIPY 493/503 (green) DNA stained with DAPI (blue), TET1 (red) (**b**), and intracellular TG content were compared (**d**)

Since TET1 had been previously shown to be inhibited in both in vivo and in vitro NAFLD models, we then explored the role of complete deficiency of TET1 (TET1 knockout) in hepatic steatosis. After 12 weeks of HFD feeding, both WT and TET1−/− mice exhibit fatty liver, showing abundant microscopic and macrovesicular steatosis by histopathology examination (Fig. 2 d, e, f). The knockout of TET1 could aggravate hepatic steatosis compared with WT mice (Fig. 2 d, e, f), which was consistent with body weight (Fig. 2 g), intrahepatic triglyceride (TG) (Fig. 2 h), serum TG (Fig. 2 l), cholesterol (Fig. 2 k), aspartate aminotransferase (AST) (Fig. 2 n) and alanine aminotransferase (ALT) (Fig. 2 o), all of which were significantly higher in TET1−/− mice. In addition, being fed 12 weeks of normal diet, the body weight of TET1−/− mice was not significantly different from that of WT mice (Fig. 2 g), however, serum TG, cholesterol, and intrahepatic TG showed significant differences. Moreover, insulin secretion and glucose tolerance were inhibited in TET1−/− mice (Fig. 2 p, s) after 12 weeks of HFD, but the insulin sensitivity was not significantly different compared to WT mice (Fig. 2 q, r, t). A similar situation was also observed in vitro. Inhibition of TET1 expression by siTET1 in both L02 and HepG2 cells increased intracellular TG (Fig. 3 a, b). The transfection of TET1 overexpression plasmid could reduce the accumulation of intracellular TG caused by FFA (Fig. 2 c, d).

### The lack of TET1 does not affect fatty acid synthesis and uptake

**Fig. 4:**
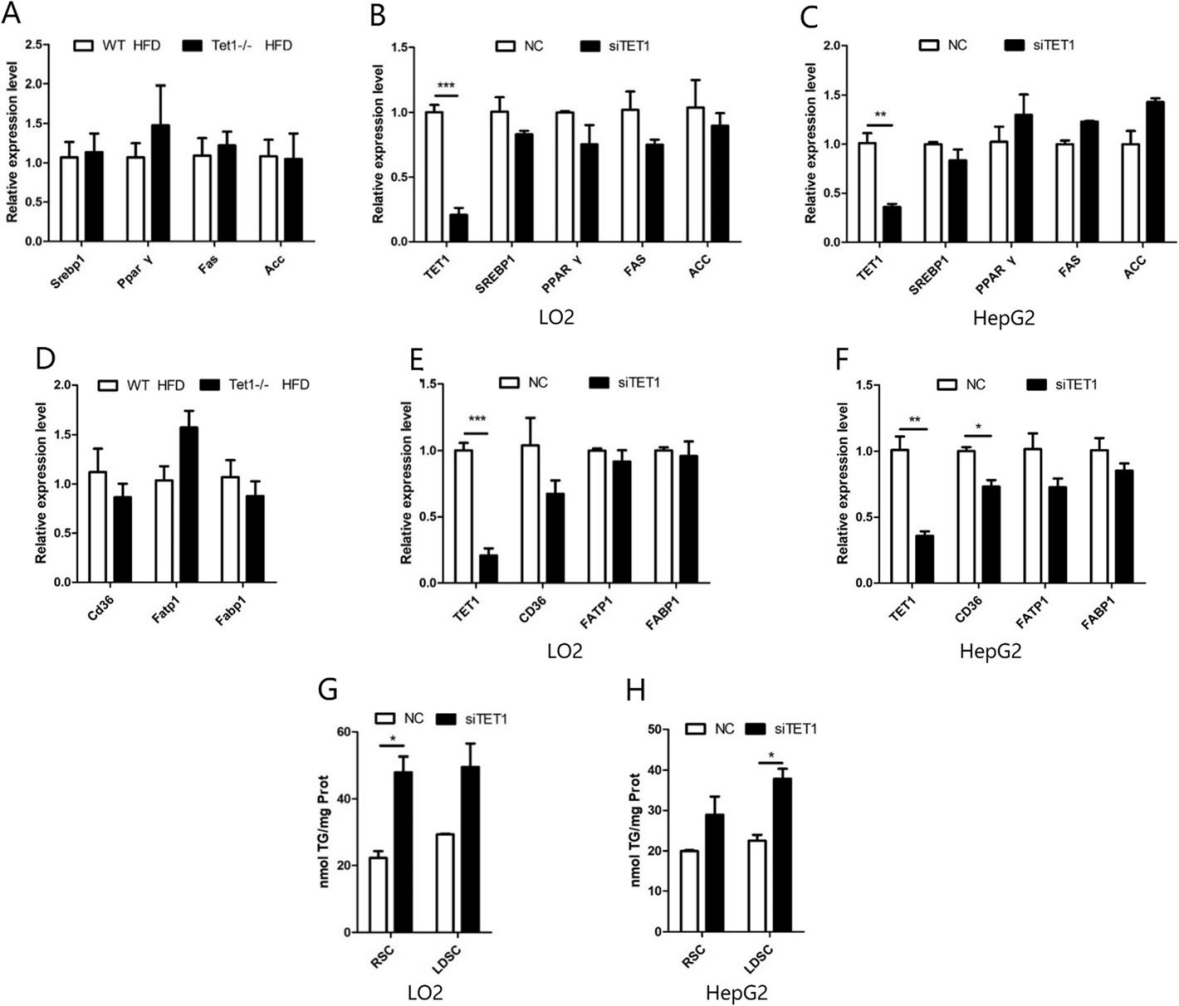
The mRNA of the fatty acid synthesis key genes in the liver of TET1-KO mice and WT mice and the L02 and HepG2 cells transfected with siRNA were detected by Q-PCR and normalized to β-actin (**a,b,c**). The mRNA of the fatty acid uptake key genes in the liver of TET1-KO mice and WT mice and the L02 and HepG2 cells transfected with siRNA were detected by Q-PCR and normalized to β-actin (**d,e,f**). After transfection of siTET1 and the control group, the cells were cultured in MEM medium containing 10% regular Serum (Regular Serum Condition; RSC) or Lipoprotein Depleted Serum (Lipoprotein Depleted Serum Condition; LDSC), and the intracellular TG content was measured (**g,h**)

**Fig. 5:**
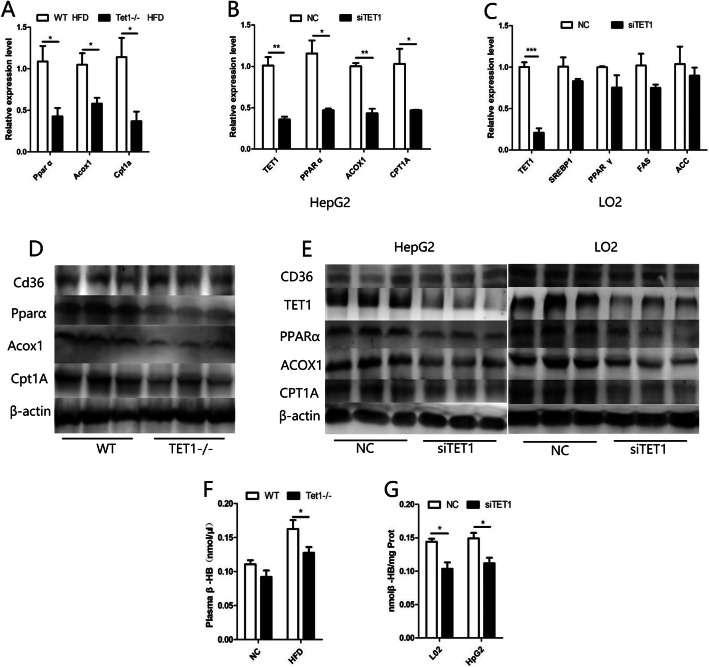
Total mRNA was extracted from the livers of HFD-fed TET1-KO and WT mice and mRNA levels were determined by Q-PCR and normalized to β-actin. The mRNA of key genes for fatty acid oxidation in the liver of TET1-KO mice and WT mice and the L02 and HepG2 cells transfected with siRNA were detected by Q-PCR and normalized to β-actin (**a,b,c**). Western blot of PPARα, ACOX1, CPT1A and CD36 in TET1-KO mice and WT mice and the L02 and HepG2 cells transfected with siRNA (**d,e,f**). The content of β-HB in the plasma of TET1-KO mice and WT mice was fed by HFD. The content of β-HB in the supernatant of cells after transfection of siTET1 or control siRNA (**f,g**)

In order to study how the lack of TET1 aggravates fatty liver caused by HFD, we first investigated several key regulators in the process of fat production. Fatty acid synthase (FAS) and Acetyl CoA carboxylase1 (ACC1) are the rate-limiting enzymes for fat synthesis, which are both regulated by the transcription factors PPARγ (peroxisome proliferators-activated receptors γ) and SREBP1(Sterol-regulatory element binding proteins 1). However, there was no significant changes in the mRNA level of these genes in the liver of TET1−/− mice compared with WT mice (Fig. 4 a). In addition, we also evaluated the effect of TET1 on fatty acid uptake by detecting the mRNA levels of relevant genes in the liver, such as CD36, FATP1 andFABP1. The results showed that FATP1 and FABP1were not significantly different except for a slight decrease of CD36 in TET1−/− mice (Fig. 4 d), while there was no significant change in the protein level of CD36 (Fig. 5 d). To confirm our findings in vitro, we inhibited TET1 expression by siTET1 in both L02 and HepG2 cells. Similar to in vivo results, no significant change in mRNA levels of fatty liver synthesis and fatty acid uptake genes was noted after TET1 inhibition (Fig. 4 b, c, e, f). In order to prevent the impact of cellular uptake of fatty acids, lipoprotein-free fetal bovine serum was used. It was shown that even after the block of cellular uptake of fatty acids, the inhibition of TET1 still promoted the accumulation of intracellular triglycerides, which also confirmed that TET1 did not affect the uptake of fatty acids (Fig. 4 g, h).

### Shortage of TET1 inhibits fatty acid oxidation pathway

The oxidation of fatty acids is mainly processed through β-oxidation pathway, which produces acetyl CoA. Acetyl CoA undergoes a series of reactions to produce ketone bodies (β-hydroxybutyric acid, acetone and acetoacetic acid), among which β-hydroxybutyrate (β-HB) accounts for the highest proportion of about 70%. The content of β-HB can indirectly reflect the case of β-oxidation of fatty acids. Thus, we examined the β-HB content in mouse plasma and cell lines to assess whether TET1 affects β-oxidation. The β-HB in the fasting plasma of HFD-fed TET1−/− mice was significantly lower than that of the control group (Fig. 5 f). Similarly, the use of siTET1 inhibited the production of β-HB in cells (Fig. 5 g). In addition, after being fed with HFD, the mRNA levels of PPARα (peroxisome proliferators-activated receptor α), ACOX1 (Peroxisomal acyl-coenzyme A oxidase) and CPT1A (Carnitine Palmitoyltransferase 1A), which are key genes in the β-oxidation pathway of fatty acids, were significantly lower in TET1−/− mice than those in WT mice (Fig. 5 a). Concurrently, their protein levels were consistent with the trend of mRNAs (Fig. 5 d). These findings were also confirmed in cell lines. After the inhibition of TET1 by siTET1, the above three key genes showed significant decrease in mRNA and protein levels (Fig. 5 b, c, e).

### TET1 affects fatty acid oxidation by hydroxymethylation of PPARα

**Fig. 6:**
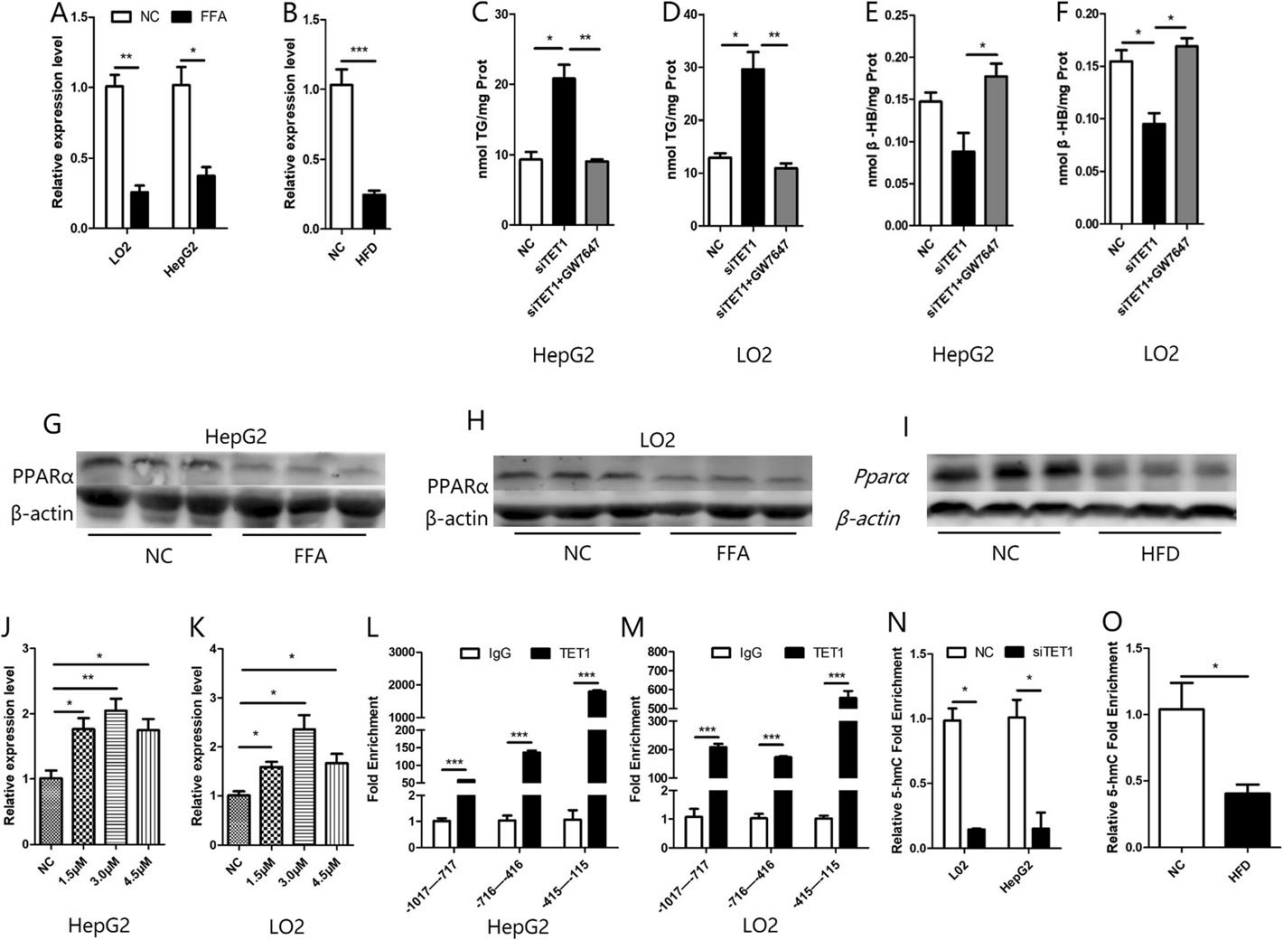
The mRNA of PPARα was detected by Q-PCR (**a,b**), and the protein of PPARα was detected by western blot (**g,h,i**), in the in vivo and in vitro models of NAFLD. After transfection of siTET1, cells were treated with a PPARα agonist to measure intracellular TG content and β-HB content in the cell supernatant (C,D,E,F). After treatment with different concentrations of methylase inhibitor 5-AZA, mRNA of PPARα was detected by Q-PCR (**j,k**). ChIP experiments confirm that TET1 binds most strongly at PPARα promoters(− 415--115) in L02 and HepG2 cells (**l,m**), hMeDIP-Q-PCR test of the 5hmC enrichment of PPARα promoters(− 415--115) in L02 and HepG2 cells, after transfection of siTET1 (**n**), hMeDIP-Q-PCR test of the 5hmC enrichment of PPARα promoters(− 208--44) in mice after HFD feeding (**o**)

PPARα is a very important transcription factor in lipid metabolism. It binds to the peroxisome proliferator response element (PPRE) located upstream of target genes, which are mainly ACOX1 and CPT1A in fatty acid oxidation [13]. We detected the expression of PPARα in NAFLD models and found that the mRNA and protein levels of PPARα in the model groups were lower than those in the control group (Fig. 6 a, b,g, h, i). Furthermore, the effect of siTET1 on inhibiting β-HB production and increasing TG accumulation was weakened after using the agonist of PPARα, GW7647 (Fig. 6 c, d, e, f). Since there is a 1932 bp CpG island starting from the − 1017 position of PPARα promoter, ChIP was used to verify the direct binding of TET1 in three segments of PPARα promoter. As a result, it was found that TET1 could directly bind to the three segments, among which the strongest binding region was − 415 to − 115 bp (Fig. 6 l, m). To further confirm that PPARα may be regulated by TET1 mediated hydroxymethylation, we first confirmed that PPARα can be regulated by methylation. After treatment of cells with different concentrations of decitabine (5-Aza-2′-deoxycytidine, a DNA methyltransferase inhibitor), the mRNA of PPARα was significantly increased (Fig. 6 j, k). Then we used the hydroxymethylation DNA immunoprecipitation (hMeDIP) kit to detect the hydroxymethylation rate of the − 415--115 bp region of PPARα promoter, which binds most strongly to TET1. The results showed that the hydroxymethylation rate of the − 415--115 bp region was significantly decreased after inhibition of TET1 (Fig. 6 n). Furthermore, there is a 1255 bp CpG island starting from the − 952 mouse position of PPARα promoter. Detection of hydroxymethylation from − 208 to − 44 in PPARα promoter showed that the rate of hydroxymethylation in the PPARα promoter of HFD-fed mice was also lower than that in the NC group. (Fig. 6 o).

## Discussion

In the present study, we discovered that TET1 expression was reduced in the in vitro and in vivo models of NAFLD. TET1 is an important hydroxymethylase that activates the expression of multiple genes and plays an important role in development and tumor. However, its role in NAFLD has been poorly understood. We have demonstrated in both in vivo and in vitro experiments that the lack of TET1 can cause an increase in the accumulation of triglycerides in hepatocytes. TET1 can promote the expression of PPARα in the methylated PPARα promoter region and increase the expression of key enzymes in the downstream fatty acid β oxidation to promote fatty acid β-oxidation, thereby inhibiting the accumulation of triglycerides in the liver.

DNA methylation, an important part of epigenetics, has received increasing attention in the pathogenesis of NAFLD [14]. The DNA promoter methylation level of PPARγ coactivator 1α (PGC1-α) in the liver of NAFLD patients was significantly higher. The methylation level of PGC1-α is also associated with fasting blood glucose, fasting insulin levels, peripheral insulin resistance, and the homeostasis model assessment of insulin resistance (HOMA-IR) [15]. These suggest that both PGC1-α transcriptional activity and insulin resistance are associated with DNA methylation. Because mitochondria are closely related to oxidative stress and reactive oxygen species (ROS), mitochondrial dysfunction can continue to produce ROS during the progression of simple steatosis to NASH. There is evidence that mitochondrial DNA methylation can also change during the course of NAFLD [16]. The TET family, as the first step in the process of demethylation and a key enzyme of hydroxymethylation [16], has been studied by many scholars for its role in the physiological [17, 18] and pathological processes of tumor development [19–21]. However, its role in the pathogenesis of NAFLD is still poorly understood. Our study showed a significant change in TET1 expression in both in vitro and in vivo models of NAFLD, while TET2 and TET3 did not change significantly.

Key genes of various fatty acid oxidation reactions are regulated by PPARα expression [13], and targeting PPARα to regulate its expression and transcriptional activity has become a new strategy for the treatment of NAFLD [22]. In the past, the fibrate drug for the treatment of hyperlipidemia is targeted at PPARα [23, 24]. However, its efficacy in the treatment of NAFLD is poor [25]. Therefore, new types of PPARα agonists have been developed for the treatment of NAFLD [26]. There are also clinical trials for new PPARα agonists in the treatment of NAFLD, but the efficacy remains to be confirmed [27]. Previous studies have confirmed that methylation of the promoter region of PPARα can affect its expression. Pregnant mice fed with a protein-controlled diet can significantly reduce the methylation level of PPARα in the offspring, while the expression of PPARα is significantly increased [28]. Furthermore, hypermethylation of the promoter regions of PPARα and CTP1A genes was shown in a model of high fructose-induced metabolic syndrome in rats [29]. In the mild and severe NAFLD cohort, PPARα and PPARδ showed significant specific site hypermethylation in patients with severe NAFLD [30]. These studies all suggest that PPARα is regulated by methylation, but no studies have shown whether PPARα is regulated by hydroxymethylation. Our study confirmed that the promoter region of PPARα can be hydroxymethylated by TET1, which indicates that PPARα is regulated not only by methylation but also by hydroxymethylation.

The 5-mC oxidation products (5-hmC, 5-hydroxymethylcytosine, 5-formylcytosine, 5-fC and 5-carboxylcytosine, 5-caC) catalyzed by the TET family are not suitable for DNMT1 substrate, so the three 5-mC oxidized derivatives are resistant to DNMT1-mediated maintenance of methylation [31, 32]. This also suggests that TET1 and 5-hmC may serve as potential new targets for the treatment of NAFLD, which may have a better prospect than simply inhibiting methylation. The level of hydroxymethylation at specific sites of PPARα may also be a new biomarker for determining the progression and prognosis of NAFLD.

## Conclusions

Our study revealed the relationship between TET1, 5-hmC, PPARα, and NAFLD, which enriched the pathogenesis of NAFLD and provided new ideas for the diagnosis and treatment of NAFLD.

## Data Availability

The datasets used and/or analysed during the current study are available from the corresponding author on reasonable request.

## Abbreviations

NAFLD: Non-alcoholic fatty liver disease
HCC: Hepatocellular carcinoma
NASH: Non-alcoholic steatohepatitis
5mC: 5-methyl cytosine
5-hmC: 5-hydroxymethylcytosine
α-KG: α-ketoglutarate
HO: Homozygous
HFD: High-fat diet
NC: Normal chow
GTT: Glucose Tolerance Test
ITT: Insulin Tolerance Test
TG: Triglyceride
TC: Cholesterol
HRP: Horseradish peroxidase
WT: Wild type
AST: Aspartate aminotransferase
ALT: Alanine aminotransferase
FAS: Atty acid synthase
FAS: Fatty acid synthase
ACC1: Acetyl CoA carboxylase1
PPARγ: Peroxisome proliferators-activated receptors γ
SREBP1: Sterol-regulatory element binding proteins 1
β-HB: β-hydroxybutyrate
ACOX1: Peroxisomal acyl-coenzyme A oxidase
CPT1A: Carnitine Palmitoyltransferase 1A
PPRE: Peroxisome proliferator response element
hMeDIP: Hydroxymethylation DNA immunoprecipitation
ROS: Reactive oxygen species
